# Chemistry

**Published:** 1886-12

**Authors:** Charles L. Steel

**Affiliations:** Demonstrator Operative Dentistry, University of Maryland.


ARTICLE III.

### CHEMISTRY.

BY CHARLES L. STEEL, M. D., D. D. S.,
Demonstrator Operative Dentistry, University of Maryland.

(Read before Virginia State Dental Association, at Natural Bridge, August 10th, 1886.)

I do not like apologies for papers, they generally imply that the author could do better under different circumstances, and often the inference is unjust.

But really, gentlemen, I, with my colleagues on your committee, have unfortunate subjects. You know that each and every one of you prepared yourselves to be bored, as soon as you saw me take my stand to talk on chemistry. We do not come to these Associations to be teased with long, tedious papers on abstruse subjects. We want something practical, something with a point and straight to the point.

It is not easy to prepare such an essay on chemistry, but the subject is not my choice. It was imposed upon me by our worthy President, with the reminder that it was customary to "pass the subject," and the hope that I would see that such was not the case in the present instance. Unfortunately, few dentists profess to know anything of chemistry—still fewer *do* know anything of it. Each year I am asked the question by students, "Why need we pay so much attention to chemistry, a study which we will have no use for in practice?" The history of chemistry itself would furnish an answer to the question. In olden times, the chemist of the day spent long and weary hours, and often sacrificed his lifc in experiments, the chief end and aim of which is set forth in the three following articles of their faith:

First.—"There exists a preparation, solid in form and red of color, called the philosopher's stone, the grand elixir, (major magisterium) the red tincture of which, when it is placed in very small quantities on melted liquid silver, mercury, lead or other common metal, causes a transmutation of the same into gold."

Second.—"The same preparation, used in very small doses as a medicine, cures all diseases, rejuvenates the old and prolongs life; wherefore it is called the panacea of life, and, since it contains the essence of gold, *aurum potabile.*"

Third.—"There is another preparation of a white color, called the stone of the second degree, the little elixir, *minor magisterium*, the white tincture, which is equal to the first in half a degree of perfection, and changes the common metals into silver."

These things were myths—the dream of the alchemist was never realized, but the discoveries made in the course of their labors founded one of the most valuable sciences.

And so with us, even though we can get along without an intimate knowledge of chemistry, were we more familiar with it, might we not bend many of its principles to our service? Doubtless our friend, Dr. Starr, can best tell us how difficult it has been to introduce electrical apparatus among dentists, to a great extent because of their ignorance of sufficient chemistry to understand the working of batteries, etc. How many new combinations might not chemical research discover to us; how many oral mysteries would be cleared up, and how much oftener would we practice intelligently rather than experimentally, did we but know more about the methods of analyzing the fluids of the mouth. I said that a paper to be read before a society should contain practical points; perhaps some one thinks I have so far introduced none, but if I may have induced anyone to take more interest in the fascinating study of chemistry, I shall consider that I have at least made one point.

I wish now to take up a special branch of chemistry, metallurgy, and under this head to call attention particularly to some things concerning amalgams.

Of course, strictly speaking, only the *analytical composition* of amalgams would come within my sphere, but I cannot refrain from saying a few words concerning its use. The lamented Dr. Webb has said, "Gold, *properly used*, is the *best* known material for the permanent preservation of the teeth." With this statement I thoroughly agree. He further says: "A cavity that can be satisfactorily filled with anything, is worth filling with gold." Granted—and yet, although I hold it as a rule, with very few if any exceptions, that gold *can* be inserted *perfectly anywhere*. I say further that there are times and places when it is the part of wisdom to use amalgam.

The first indication for its use which I would mention is the cheapness with which it may be manipulated. This may seem a sordid motive, but there are patients who would willingly pay the prices asked for amalgam, and thereby save their teeth indefinitely, who would be frightened, if not out of their wits, certainly out of our chairs, at the mere mention of the *necessary* charge for gold, and while those who practice dentistry for fun may do it, yet those who practice for a living cannot, in justice to themselves, spend hours upon troublesome operations without being suitably compensated, which means compensated beyond the ability of many of our patients. Again, we find patients physically so weak that it would be a species of unjustifiable persecution to subject them to so great a tax upon their nervous system as extensive contouring with gold demands.

And a third reason—it may be *possible* to insert gold perfectly anywhere, yet not *everybody* can do it. Repeated failures in certain classes of cases should have taught some men that they are *not* of the number who can; these men can be *honest* by using amalgam, but not otherwise.

There may be some men who *can* put gold anywhere perfectly, but even the best fail sometimes, even we Virginia dentists have been known to fail "semi-occasionally," and these failures occur so much more frequently in posterior-approximate cavities of molars that it will often become a question as to whether the chances are not in favor of a safer operation being done in these positions with amalgam than with gold. Now, what are the objections to amalgams? First, they are of an objectionable color, and become still more unsightly with age, from being discolored by the fluids of the mouth. Next, almost all of the makes on the market shrink, even though we may occasionally get an amalgam which seems to stand the shrinkage test perfectly. I still say we cannot trust to amalgam for a perfect filling, because so many things influence the behavior of alloys that the same formula may give different results at different times because of some difference in the process of melting, some undetected impurity in the metals used, or even difference in the age of the alloy. From the foregoing remarks we can see what are the points to be sought after in amalgams, viz., after the necessary strength is obtained, we wish to obtain an amalgam which will not shrink and which will longest retain a good color in the mouth. These results can only be secured by experiment, for so singular, and oftimes so unexpectedly different from the character of the ingredients, are the characteristics of the resulting alloy, that no reliable rules can be based on the characteristics of the metals which it is proposed to use. A striking example of this fact is seen in alloying gold, a metal too soft to use pure for many purposes, with platinum, a metal softer even than gold and more pliable, the resulting compound is hard and very elastic. Neither gold nor platinum is soluble in anything but nitro-muriatic acid, but an alloy of these metals is readily soluble in nitric acid. Amalgam hardens by crystallization, or the arrangement of its particles into distinct geometrical figures, in this process some combinations will expand, others shrink, hence an alloy should have its constituent metals combined in such proportions that the various metals will so counter-balance each other as to prevent either shrinkage or expansion of its compounds. The bulk of all amalgams consists of about equal parts of silver and tin—the differences in amalgams are due to slight variations in the above mentioned proportions of silver and tin and to the addition of small per cents of various other metals. In proportion as the silver is in excess is the alloy more difficult to amalgamate, requiring more mercury and more time for mixing, the more rapidly does it set, and the harder it is after setting, it discolors more readily and the shrinkage is less, and vice versa, when the tin is in excess, our alloy amalgamates more readily, sets more slowly, is softer after having set, discolors less readily, but shrinks more.

Of all the metals which are added to alloy, none exert a more beneficial effect than zinc. From five to seven per cent. of this metal almost entirely controls the shrinkage of the silver and tin, while the more zinc we add the better will our alloy resist the action of discoloring agents. An excess, however, causes expansion. Unfortunately, however, our alloy as it now stands—silver, tin, zinc—is not sufficiently hard to withstand mastication ; the zinc, in fact, although improving it as far as shrinkage and color are concerned, tends rather to deteriorate the compound, as far as strength is concerned.

It has been claimed that amalgams containing zinc would frequently cause pains in the tooth, and this trouble was laid upon the back of that dentists' scape-goat, galvanic action. Just here, let me say that the theory of galvanic action between the metals of fillings, is something in which I have very little faith, so little that I do not permit it to influence my practice in the least. I have only seen a *very few* instances of trouble after metal fillings, which could not be accounted for in a more reasonable way, and these cases were so obscure that I am not, by any means, sure that alvanic action explained the trouble.

I think that such troubles are always due to the thermal shocks which are so readily transmitted through any metallic filling, or else to the irritating effect which any metal sometimes seems to exert when applied to freshly cut surface of dentine, especially in very young and sensitive teeth; and the trouble can therefore be avoided by using a liberal layer of gutta-percha under the amalgam, or else by filling the entire cavity with gutta-percha, temporarily. But to return to our alloy. We left it with a body of silver and tin, with sufficient zinc to control shrinkage and improve color, but lacking in strength. Now this element can be given by two metals, each of which, however, has its disadvantage. First, I mention copper: this improves the strength wonderfully, an addition of two to five per cent. making a very hard amalgam; it increases the facility with which the alloy amalgamates, and when amalgamated the mass has a most soft, plastic feeling under the finger, and the grain is very dense; but alas, it greatly deteriorates it, as far as color is concerned, causing it to turn dark, and if much is used, quite black, in a comparatively short time. Gold is the other metal: *it* tends to harden our amalgam; though not quite to the same extent as copper. It, however, has little or no effect upon the shrinkage. It is no recommendation to an amalgam to call it a "Gold and Platina alloy." I do not mean to speak against such preparations; some of them are fairly good; but they are not so because they contain gold and platina, for gold exerts little effect, except to slightly harden our amalgam, and, possibly, slightly to facilitate amalgamation, while platinum has little effect. I have mentioned the metals which are chiefly employed to advantage in amalgams. Many others are used, but they are of little, if any, practical value; their chief use being to make one alloy different from another, or as a handle for advertising. Even though we may get an alloy *capable* of giving fine results, we can greatly alter its behavior by our own treatment of it. Mercury is often impure because containing other metals. If we are not careful to have a refined mercury, we may change the whole nature of our alloy by introducing this new metal. The re-distilled mercury of the S. S. White Dental Manufacturing Company, is practically pure, and those who cannot obtain this, can sufficiently refine commercial mercury by agitating it in hydro-chloric acid, or nitric acid—one part acid to two or three parts of water.

Now; in preparing our amalgam, it is very important to secure the right proportions of mercury and alloy; an excess of mercury left in the amalgam tends to make it soft and porous, and in pressing out an excess we are apt to deteriorate the amalgam. This is doubtless due to the fact that mercury, having greater affinities for some metals than for others, brings away with it some portions of some of the metals; thus altering the proportion which had been found to give the best results. All amalgams have the surfaces of their particles more or less oxydized by exposure. For this reason it is best that it should be kept in a tightly stoppered vial. This oxide, incorporated in the amalgamated mass, is a decided disadvantage, besides rendering the alloy difficult to mix with the mercury. A few drops of diluted sulphuric acid poured upon the amalgam, while working it, will take away the oxide and cause the alloy to amalgamate with remarkable rapidity. After using the acid, the amalgam should be washed in aqua ammonia, or a solution of bicarbonate of soda, to remove all traces of the acid. Alcohol answers a very good purpose for washing amalgam, but is not so effective as the process above mentioned.—*Southern Dental Journal.*

